# Supplementary figures and images for: Myo-REG: A Portal for Signaling Interactions in Muscle Regeneration
Source: Front Physiol. 2019 Sep 27;10:1216. doi: 10.3389/fphys.2019.01216 (PMC6776608; doi:10.3389/fphys.2019.01216)

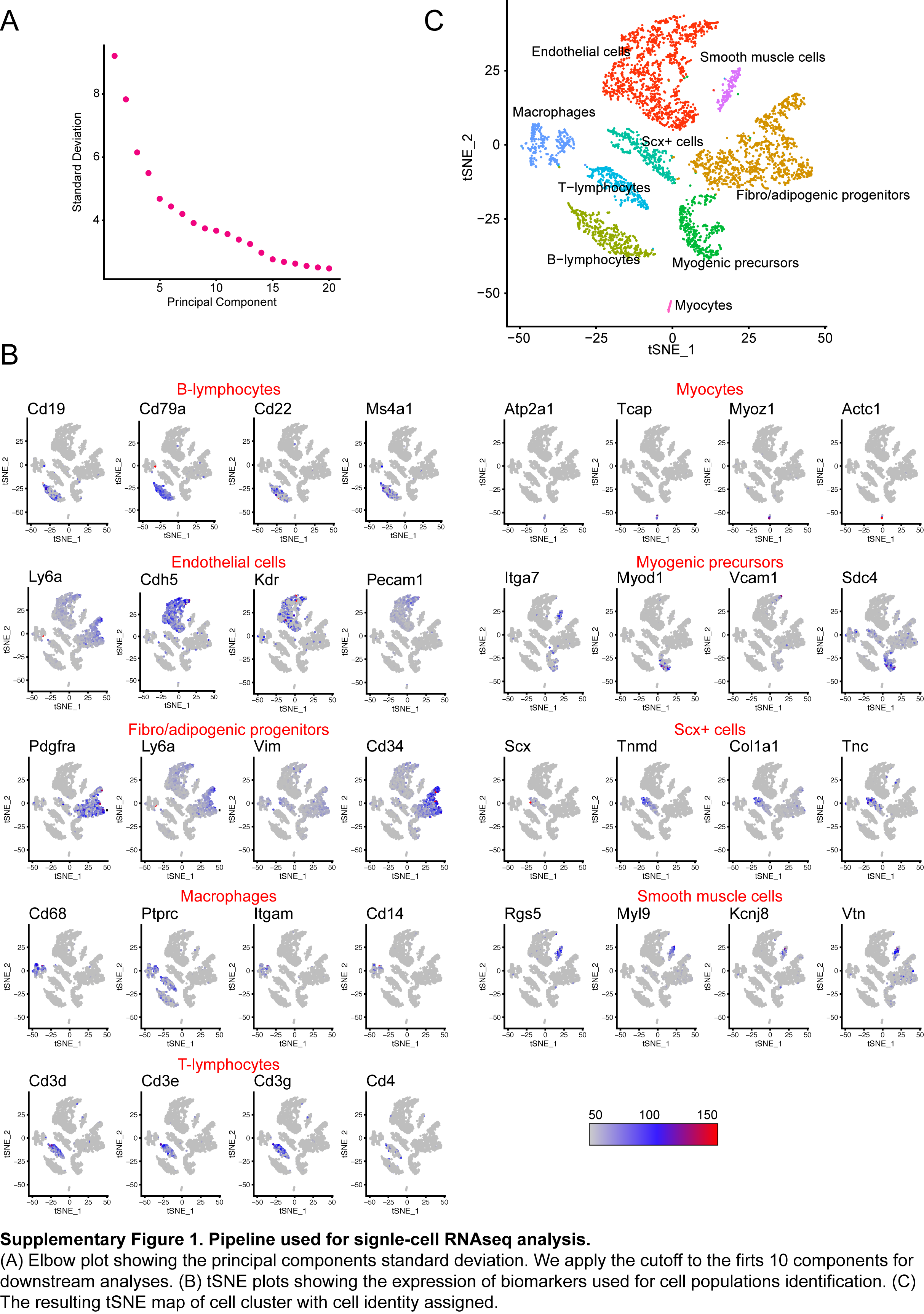

Supplement: Supplementary file 1 [file Image_1.TIF]
